# Control of Bovine Leukemia Virus in Three US Dairy Herds by Culling ELISA-Positive Cows

**DOI:** 10.1155/2019/3202184

**Published:** 2019-06-25

**Authors:** Vickie J. Ruggiero, Paul C. Bartlett

**Affiliations:** Department of Large Animal Clinical Sciences, College of Veterinary Medicine, Michigan State University, East Lansing 48823, USA

## Abstract

The objective of this trial was to evaluate a test-and-cull approach to controlling bovine leukemia virus (BLV) in US dairy herds with a low BLV prevalence. Despite worldwide distribution of the virus, 21 nations have eradicated BLV from their dairy cattle and are currently considered ‘BLV-free.' In contrast, the US has attempted no industry-wide BLV control programs and has experienced an increase in BLV prevalence among dairy cows to about 40%. This raises concerns about production efficiency, herd health, and sustainability. In a pilot field trial with three Midwestern-US dairy herds, a test-and-cull approach using ELISA screening of milk samples was successful in reducing BLV prevalence in two herds. In the third herd, BLV prevalence increased following the introduction of infected heifers that were raised at an out-of-state calf raising facility. This trial demonstrated that a test-and-cull approach to BLV control can be successful in US dairy herds with low BLV prevalence, but ongoing surveillance is necessary to prevent reintroduction of the virus.

## 1. Introduction

Currently, 21 nations have eradicated BLV from their dairy herds and others have implemented eradication programs [1–3]. Eradication has been achieved by testing blood or milk for BLV antibodies, followed by culling of the animals that test positive. Occasionally, BLV-antibody positive animals were temporarily segregated from the rest of the herd until they could eventually be culled. These programs were primarily implemented in countries with low BLV prevalence [4]. The objective of our study was to evaluate the efficacy of this approach in US dairy herds with a similarly low initial BLV prevalence.

The prevalence of BLV in US dairy cattle has been increasing over the past decades, with the most current report estimating that 94% of herds and 42% of dairy cows are positive for BLV antibodies [5] using a lactation-stratified 40-cow sample termed a “BLV Herd Profile” [6]. Although about 6% of the herds in this study were BLV-free, this is lower than USDA-NAHMS reports that found ~16% and a 2010 Michigan study that found 13% of herds were free of BLV [7–9]. This observation supports the theory that herds which eliminate BLV should be able to keep their herds from being reinfected if they are careful to not introduce infected animals.

Historically, the major economic impetus for BLV control has been the prevention of lymphoma (lymphosarcoma), as other impacts of BLV have only recently been recognized. Lymphoma affects an estimated 5% of infected cattle [10, 11], and in the US, lymphoma is the most common reason for postmortem carcass condemnation at slaughter [12]. However, approximately 30% of ELISA-positive cattle have a lymphocytic leukosis (lymphocytosis) which is accompanied by immune suppression [13–15]. This immune dysfunction may be the cause for the epidemiologic observations that BLV-antibody positive cows have decreased milk production [5, 16, 17] and a shortened lifespan [17, 18]. A 2003 economic analysis found the annual cost of BLV to the dairy industry to be $525 million lost annually [19] and a more recent, informal estimate by our research group showed that the cost to the W.K. Kellogg Biologic Station Pasture Dairy Center was $379.92 per infected cow yearly [20, 21]. In comparison, mastitis costs to the dairy industry have been estimated at $450 per case [22, 23] or $200 per milking cow annually [24]. Another impact of BLV is that the export of US animals and animal products has become more difficult, with some countries such as those within the EU requiring that animals come from BLV-free herds and be tested for BLV prior to introduction [25]. Epidemiologic studies have shown an association between BLV infection and infectious conditions such as mastitis, respiratory diseases, and gastrointestinal diseases, as well as delayed recovery from some infections [26], making animal welfare issues a concern both due to the risk of lymphoma and from immune dysfunction. Public health issues related to BLV are still being investigated, and public perception of the dairy industry could be impacted by these reports [27, 28]. Taken together, there is a strong case for controlling BLV in the US. Therefore, we designed a pilot BLV control program to determine if dairy herds under US management conditions with a <5% prevalence of BLV antibodies could achieve eradication by periodic BLV ELISA testing of milk samples followed by selectively culling or segregating antibody-positive cows.

## 2. Materials and Methods

### 2.1. Herd Enrollment and Study Design

Herd enrollment requirements were as follows: (1) BLV-antibody prevalence by ELISA ≤5% and (2) herd managers who were willing to cull ELISA-positive cattle or at least temporarily segregate them for eventual culling. All lactating cows in the milking herd were tested by milk ELISA at least yearly and sometimes dry cows and young stock were tested by serum ELISA prior to entering the milking herd. The timing of BLV testing was customized to each herd's management practices, resources, and level of engagement, with the goal of minimizing the amount of time an identified antibody-positive cow might be in contact with BLV-susceptible herd mates. Results of ELISA testing were immediately given to the herd managers, whom we encouraged to cull the cows that were positive for BLV antibodies or separate them from BLV-negative animals if they could not be immediately culled.

#### 2.1.1. Herd “M”

Herd M was a free-stall dairy with 150 milking cows at enrollment in August 2015. Individual-cow milk BLV ELISA testing was carried out approximately every six months. Additional tests were conducted on milk samples from cows that were dry at the semiannual testing day, or on cows that we expected to be dry at the upcoming semiannual test day, and from freshened (1st lactation) heifers that had entered the milking herd between semiannual tests, as described in Table 1.

#### 2.1.2. Herd “R”

Herd R was a free-stall dairy with 850 milking cows at enrollment in July 2015. Individual-cow milk BLV ELISA testing was carried out yearly. Additional tests were conducted on milk samples from freshened (1st lactation) heifers and cows that had been dry at the previous annual test, as described in Table 2.

#### 2.1.3. Herd “S”

Herd S was a free-stall dairy (except for transition heifers that were on bedded pack) with 350 milking cows at enrollment in December 2014. Individual-cow milk BLV ELISA testing was carried out yearly. Additional tests were conducted on milk samples from cows that were dry at the annual test day, or that were going to be dry at the upcoming annual test day, and from freshened (1st lactation) heifers that had entered the milking herd between semiannual tests. The herd managers were highly motivated to control BLV and conducted additional interim serologic testing of dry cows and heifers and testing of bulk tank milk samples as described in Table 3.

### 2.2. Blood and Milk Sample Collection

Routine milk samples were collected by DHI technicians into containers with bronopol/natamycin preservative and transported to a NorthStar Cooperative, Inc. Laboratory (Grand Ledge, MI, USA or Kaukauna, WI, USA). Milk samples were analyzed for milk components (fat, protein, somatic cells, etc.) first and then evaluated for BLV antibodies via ELISA. If the cow to be tested was not lactating, blood samples were collected into clot activator/polymer gel evacuated tubes and transported to the NorthStar Cooperative Michigan Laboratory for testing. The animal procedures for this study were reviewed and approved by the MSU Institutional Animal Care and Use Committee.

### 2.3. ELISA Test for BLV Antibodies

A 2013 study by Walsh et al. found a near-perfect agreement between serum and milk ELISA test results for BLV using a modified ELISA kit [29]. A similarly modified ELISA (IDEXX Laboratories, Inc., Westbrook, ME, USA) for BLV antibodies [6] was performed by a NorthStar Cooperative commercial diagnostic laboratory. Aliquots of milk samples were diluted 1:30 (individual animal) or 1:2 (pooled/bulk tank). Alternatively, diluted (1:30) serum from nonlactating cows was used. Briefly, samples were added to 96-well BLV-coated ELISA plates and washed. BLV antibodies were detected by reaction with horseradish-peroxidase-labeled antibodies to bovine immunoglobulin with addition of an enzyme substrate. Reaction times were standardized by color development of positive controls, and the reaction was stopped by addition of 0.5 N H_2_SO_4_. Results were reported as corrected 450nm optical density (*OD*) measurements (raw sample OD - negative control OD). Milk samples with a corrected OD > 0.1 and serum samples with a corrected OD > 0.5 were considered positive for anti-BLV antibodies.

## 3. Results

### 3.1. Herd M

Herd M had a starting BLV prevalence of 2.2% and culled all known BLV-antibody positive (“positive”) cows after their first test of all lactating cows in the herd in August 2015 (Table 1). The testing protocol for this herd was approximately semiannual milk testing of all lactating cows, with additional tests of cows that were newly lactating since the last semiannual test, i.e., cows that had been dry at the semiannual test and 1st lactation heifers that entered the milking herd after calving.

The first additional test in November 2015 identified one positive cow. This animal was still in the herd at the semiannual test of all milking cows in February 2016 and was the only positive animal identified. It was subsequently culled in March 2016. For the remainder of the intervention, the only positive animals identified were 1st lactation heifers, one at each semiannual test, that had recently entered the milking herd.

### 3.2. Herd R

Herd R had a starting BLV prevalence of 3.2% in July 2015 (Table 2). The herd manager culled some positive animals from the herd and moved any remaining positive animals to a single pen to segregate them from the negative cattle. The testing protocol for this herd was similar to Herd M, semiannual milk tests of all lactating cows, with additional tests of cows that were newly milking since semiannual tests.

The first interim test in September 2015 identified five positives, all of which were 1st lactation heifers. At the next test of all lactating cows in February 2016, BLV prevalence had increased to 3.67% with 31 positive cows. Of these, fourteen were 1st lactation heifers, twelve were positive at a prior test, and five previously BLV-antibody negative cows were now positive. An interim test of 1st lactation heifers in May 2016 identified a 10% prevalence in this age group and the herd manager indicated that they were no longer able to move all positive animals to the segregated pen or cull them. The BLV prevalence at the semiannual test of all lactating cows in August 2016 increased to >5%. At this point, the herd was no longer within the inclusion criteria for the study and the August 2016 test concluded their participation.

### 3.3. Herd S

Herd S had a starting prevalence of 2.3% (Table 3) in December 2014 and culled all positive animals shortly after testing. The herd manager wanted to be proactive, so nearly 70 cows and heifers that were due to enter the milking herd after calving in the next six months were tested by serum ELISA in February 2015. Two additional positives were identified and subsequently sold. The testing protocol occurred approximately on an annual basis for all lactating cows, with quarterly interim tests for cows that initiated lactation between the semiannual tests. Bulk milk tank samples were tested for BLV antibodies at unstructured intervals as an exploratory surveillance method.

Interim tests in March and July (including a July bulk tank sample) were negative for BLV antibodies, while two positive animals were identified in October 2015 and were subsequently culled. Only one animal was positive for BLV antibodies at the January 2016 milk test of all lactating cows, and one was positive at the April 2016 interim test, both of which were culled. A bulk tank sample in April 2016 was also negative. In July 2016, two positive animals were identified at the interim milk test, and the bulk tank sample was positive. Both positives were culled. We conducted serum testing of heifers due to calve in the upcoming six-month period in September 2016, with no positives identified. At the October 2016 interim milk test, there were three positive animals, which were then culled, and the bulk tank sample remained positive as well.

Subsequently, we began monthly serum tests of pregnant heifers with a due date within six months. In November, December, and January, two positives were identified—one in December and one in January—and sold. At the January 2017 milk test of all lactating cows, five cows were positive, as was the bulk tank sample. Notably, all five had been antibody-negative on serum tests as heifers. The January 2017 milk test was the final test of all lactating cows for this study, but we conducted the final three monthly serum tests on heifers in February, March, and April 2017, as had been agreed upon with the herd manager. Only one positive animal was identified on these tests (in February 2017) and she was sold. The herd prevalence in Herd S dropped from 2.3% to 0.3% after the first year of this program. Although the prevalence increased to 1.5% in the second year due to the first lactation heifers, it was lower compared to the starting prevalence.

## 4. Discussion

The test-and-removal approach to BLV eradication has been successful in many other nations, most of which started their control programs with low BLV prevalence [4, 25]. These programs used serum testing almost exclusively, which is inconvenient for dairy producers. Our results demonstrate that this approach also works for milking herds in the US which have similarly low BLV prevalence, primarily using milk samples already being collected for milk component testing. However, a limitation of milk testing is that the inflow of infected heifers quickly reintroduces BLV into the milking herd. US herds which can reduce their prevalence to less than 5% may consider eradicating BLV from their herds by removing all ELSA-positive cows, but need to ensure that incoming heifers do not reintroduce the infection as was seen in the current study.

In just 19 months, Herd M appeared to nearly eradicate BLV. However, ongoing monitoring will be necessary before complete eradication can be claimed. Similarly, Herd S appeared close to having eradicated BLV from the milking herd, but incoming infected young stock prevented complete elimination during the 29 months of their participation. Herd R discovered a relatively high BLV prevalence from heifers raised off-premises. This led to a progressive increase in herd BLV prevalence when the herd manager could not cull or segregate this large number of incoming BLV-positive animals.

The results of Herd S demonstrated an additional concern for BLV eradication: that of possible latent infections, in which a BLV infection—perhaps from calf-hood—remains sequestered and dormant until later in life. This is consistent with mechanisms seen in other retroviruses that avoid immune detection [30] and the potential for latent BLV infections has been speculated about since early PCR tests were sometimes unable to detect BLV provirus in antibody-positive animals (e.g., Murtaugh [31]). Klintevall [32] reported a calf which, after experimental infection with BLV, appeared to have maintained the virus in a latent state, sequestering provirus in the spleen and having no detectable circulating antibodies. The five first lactation heifers in Herd S that were BLV-antibody positive in January 2017 had previously tested negative for BLV antibodies and no other animals in the herd were known to be BLV-positive. This suggests that these heifers had latent infections that became active after entering the milking herd. Therefore, it is clear that monitoring heifers for BLV is important both before and shortly after they enter the milking herd is an important consideration for BLV control programs.

The test and cull of all ELISA-positive cows method to control BLV in the three study herds were only economically feasible because they started the program with a low BLV prevalence. US dairy herds with BLV prevalence closer to the national average (>40%) will probably need to reduce their prevalence to a point where test and removal are economically feasible.

## 5. Conclusions

Depending on the herd, test and cull by using milk ELISA as means to reduce or eliminate BLV from dairy herds with low BLV prevalence may be effective. However, average BLV prevalence in US dairy herds is over 40%. Therefore, future BLV research is needed to determine cost-effective methods of reducing BLV prevalence to a point where test and cull may be cost-effective.

## Figures and Tables

**Table 1:** Bovine leukemia virus (BLV) antibody testing schedule and results for Herd M.

Test Date	Test Type	Description	Results^1^
August 2015	Herd (milk)	All cows in the milking herd	3/138 (2.2%)
November 2015	Additional (milk)	Cows dry at August test and fresh heifers^2^	1/42
February 2016	Herd (milk)	All cows in the milking herd^3^	1/153 (0.6%)
March 2016	Additional (milk)	Cows dry at February test and fresh heifers	0/13
August 2016	Additional (milk)	Cows projected to be dry at late August test	0/16
August 2016	Herd (milk)	All cows in the milking herd^4^	1/143 (0.7%)
October 2016	Additional (milk)	Cows dry at August test and fresh heifers	0/18
February 2017	Herd (milk)	All cows in the milking herd^4^	1/150 (0.7%)
March 2017	Additional (milk)	Cows dry at February test and fresh heifers	0/16

^1^ BLV-antibody positive (“positive”) over total tested, with point prevalence (%) for herd tests.

^2^ Excludes five cows tested in August 2015 that were inadvertently retested in November.

^3^ The one (1) positive cow was the same cow identified at the November 2015 test, subsequently culled.

^4^ The one (1) positive was a first lactation heifer, subsequently culled.

**Table 2:** Bovine leukemia virus (BLV) antibody testing schedule and results for Herd R.

Test Date	Test Type	Description	Results^1^
July 2015	Herd (milk)	All cows in the milking herd	27/849 (3.2%)
September 2015	Interim (milk)	Cows dry at July test and 1st lactation heifers^2,3,4^	5/136
February 2016	Herd (milk)	All cows in the milking herd^2,5^	31/844 (3.7%)
May 2016	Interim (milk)	Test of 1st lactation heifers^2,6^	22/218
August 2016	Herd (milk)	All cows in the milking herd^2,7^	46/849 (5.4%)

^1^ BLV-antibody positive (“positive”) over total tested, with point prevalence (%) for herd tests.

^2^ Heifers had comingled with animals from other herds at the out-of-state calf-raising facility.

^3^ All five (5) positive animals were 1st lactation heifers.

^4^ Excludes one (1) cow tested in July 2015 that was inadvertently re-tested in September.

^5^ Five (5) positive cows were new infections in previously negative animals, fourteen (14) were 1st lactation heifers, and twelve (12) were previously positive animals.

^6^ Includes ninety-four (94) 1st lactation heifers previously tested in February, eleven (11) of which were positive at that time.

^7^ Seven (7) positive cows were new infections in previously negative animals, nine (9) were 1st lactation heifers, and thirty (30) were previously positive animals.

**Table 3:** Bovine leukemia virus (BLV) antibody testing schedule and results for Herd S.

Test Date	Test Type	Description	Results^1^
December 2014	Herd (milk)	All cows in the milking herd	8/343 (2.3%)
February 2015	Interim (serum)	Cows and heifers due to calve in the next six months^2^	2/69
March 2015	Interim (milk)	Cows dry at the December test	0/10
July 2015	Interim (milk) Bulk tank (milk)	All cows and heifers that calved since the March test	0/27 Negative
October 2015	Interim (milk)	All cows and heifers that calved since the July test	2/43
January 2016	Herd (milk)	All cows in the milking herd	1/342 (0.3%)
April 2016	Interim (milk) Bulk tank (milk)	All cows and heifers that calved since the January test	1/39 Negative
July 2016	Interim (milk) Bulk tank (milk)	All cows and heifers that calved since the April test	2/31 Positive
September 2016	Interim (serum)	Heifers due to calve in the next six months	0/38
October 2016	Interim (milk) Bulk tank (milk)	All cows and heifers that calved since the July test	3/30 Positive
November 2016	Interim (serum)	Heifers due to calve in the next six months	0/10
December 2016	Interim (serum)	Heifers due to calve in the next six months	1/14
January 2017	Interim (serum)	Heifers due to calve in the next six months	1/14
January 2017	Herd (milk) Bulk tank (milk)	All cows in the milking herd^3^	5/343 (1.5%) Positive
February 2017	Interim (serum)	Heifers due to calve in the next six months	1/35
March 2017	Interim (serum)	Heifers due to calve in the next six months	0/12
April 2017	Interim (serum)	Heifers due to calve in the next six months	0/8

^1^ BLV-antibody positive (“positive”) over total tested, with point prevalence (%) for herd tests.

^2^ Excludes one (1) cow tested in December 2014 that was inadvertently re-tested in February.

^3^ All five (5) positive animals had previously tested negative on serum as heifers.

## Data Availability

The ELISA test results and survey responses collected in the course of this study have not been made available to protect the confidentiality of participating dairy producers. Aggregate data may be available from the corresponding author upon request.

## Abbreviations

BLV: Bovine leukemia virus
ELISA: Enzyme-linked immunosorbent assay
OD: Optical density
PVL: Proviral load.
